# *FeliLeish*: An Update on Feline Leishmaniosis and Factors Associated with Infection in Different Feline Populations from Italy

**DOI:** 10.3390/pathogens12111351

**Published:** 2023-11-14

**Authors:** Eva Spada, Germano Castelli, Federica Bruno, Fabrizio Vitale, Francesco La Russa, Vito Biondi, Sara Accettulli, Antonella Migliazzo, Aurora Rossi, Roberta Perego, Luciana Baggiani, Daniela Proverbio

**Affiliations:** 1 Dipartimento di Medicina Veterinaria e Scienze Animali (DIVAS), Università degli Studi di Milano, Via dell’Università 6, 26900 Lodi, Italyluciana.baggiani@unimi.it (L.B.); daniela.proverbio@unimi.it (D.P.); 2 Centro di Referenza Nazionale per le Leishmaniosi (C.Re.Na.L), Istituto Zooprofilattico Sperimentale (IZS) della Sicilia A. Mirri, Via G. Marinuzzi 3, 90129 Palermo, Italy; germano.castelli@izssicilia.it (G.C.); federica.bruno@izssicilia.it (F.B.); fabrizio.vitale@izssicilia.it (F.V.); francesco.larussa@izssicilia.it (F.L.R.); accettullisara@gmail.com (S.A.); 3 Department of Veterinary Sciences, University of Messina, Polo SS. Annunziata, 98122 Messina, Italy; vito.biondi@unime.it; 4 Dipartimento di Prevenzione UOC Sanità Animale, Igiene degli Allevamenti e delle Produzioni Zootecniche, ASL Latina, 04100 Latina, Italy; a.migliazzo@ausl.latina.it

**Keywords:** *Leishmania infantum*, IFAT, epidemiology, risk factors, protective factors, qPCR

## Abstract

Feline leishmaniosis is a worldwide infection caused by the parasite of the genus *Leishmania* transmitted by sandflies. Based on the complexity of epidemiology and diagnosis of this infection, the role of cats in the epidemiology and clinical impact of disease is still under debate. By using serological and molecular methods, this study aimed to update the epidemiology of the infection in different feline populations from various areas of Italy and to study factors associated with the infection. Of 1490 cats tested, 124 (8.3%, 95% CI 6.9–9.9) were infected, 96 had only specific *L. infantum* IgG, 18 were only positive for parasite DNA and 10 were both IFAT and qPCR positive. Risk factors for infection were sampling in the winter season (OR = 3.2, 95% CI 2.2–4.8), originating from the Sicily region (OR = 2.0, 95% CI 1.3–3.0), male gender (OR = 1.8, 95% CI 1.1–3.2), outdoor lifestyle (OR = 2.3, 95% CI 0.9–5.6) and seropositivity for FIV antibodies (OR = 2.2, 95% CI 1.2–4.2), while sampling in the spring (OR = 0.5, 95% CI 0.3–0.7) and summer (OR = 0.3, 95% CI 0.1–0.7), and originating from the Lazio region (OR = 0.1, 95% CI 0.05–0.4) were protective factors for infection. In endemic areas, *Leishmania* infection should be investigated by using both serological and molecular methods and cats should be protected from sandfly bites, particularly if they are FIV infected.

## 1. Introduction

Feline leishmaniosis is a worldwide infection caused by the parasite of the genus *Leishmania* transmitted by sandflies of the genus *Phlebotomus* or *Lutzomyia* [1,2,3]. In Europe, *L. infantum* is the causative agent of leishmaniosis in cats and sandflies of the genus Phlebotomus are the principal vectors for transmission of parasites to cats [4]. While overall there is no evidence of widespread increased incidence of autochthonous human leishmaniosis by *L. infantum* in European countries [5], the infection is still considered emergent in cats. Published case reports and epidemiological studies have reported infected cats in most European countries in the last decades [6,7,8,9,10,11,12,13,14,15,16,17,18,19], including in areas where the infection is not endemic in dogs and humans [15,16,18,20]. Integrated leishmaniosis surveillance and reporting following the One Health approach needs to be enhanced to improve disease control [5] and larger cohorts of cats and vector studies are necessary to determine whether felids may act as reservoirs or sentinels of human *Leishmania* infection [21].

In areas endemic for leishmaniosis, infection in cats is influenced by a number of different factors, in particular a cat’s immunocompetence. A number of studies have identified retrovirus coinfection as a risk factor for *L. infantum* infection in cats [12,15,22,23,24,25]. However, many other factors can be related to infection in cats and are either risk or protective factors for the infection. Infected cats can be healthy or unhealthy, with the latter showing signs not pathognomonic for *Leishmania* infection such as weight loss, lymphadenomegaly, stomatitis and dermatological or ocular signs [26,27,28,29,30,31]. Diagnosis can be challenging, not only because clinical signs are not specific, but also because some cats are positive on molecular testing but not on serological tests for *L. infantum* specific antibodies or vice versa. Due to the complex epidemiology and diagnosis of this infection in cats [32,33,34,35], the role of cats in disease epidemiology and its clinical impact is still debated. Our study aimed to update the epidemiology of the infection in different feline population from various areas of Italy, with particular focus on the risk or protective factors associated with infection.

## 2. Materials and Methods

From January 2020 to May 2023, a total of 1490 cats were investigated for *L. infantum* infection, searching for specific feline immunoglobulin G (IgG) by means of immunofluorescence antibody test (IFAT) and/or for parasite DNA by using quantitative real-time PCR (qPCR) performed on blood samples and/or on popliteal lymph node aspirate. The same cats were also tested for feline leukemia virus (FeLV) p27 antigen and antibody to feline immunodeficiency virus (FIV) target antigens p24 and gp40 using a commercial rapid enzyme-linked immunosorbent assay (ELISA) kit (SNAP^®^ Combo Plus FeLV Ag/FIV Ab, IDEXX Laboratories, Lenexa, TX, USA).

For each sampled cat, data were collected (where possible) on: date of sample collection, region of origin of the cat (Lombardy region in northern Italy, Lazio in central Italy, Sicily in southern Italy), demographic data (age, gender, reproductive status, breed, hair length), husbandry data, i.e., owned, shelter or stray colony cat, cohabitation with dogs, possibility of outdoor access, prophylaxis against ectoparasites, and results of physical examination. Cats were classified as apparently healthy or not and whether they were affected by signs compatible with feline leishmaniosis (lymph node enlargement, skin or ocular signs). Cats were grouped according to age as follows: kitten (younger than 12 months), adult (between 1 and 10 years) and senior (older than 10 years).

### 2.1. Ethical Considerations

Our study complied with current Italian legislation on research and was approved and funded by the Italian Health Minister under the name of “*FeliLeish*”. All samples from owned cats were collected for diagnostic purposes with oral informed consent obtained from the owners of cats. Samples were collected from shelter and stray cats undergoing spaying surgery for pre-operative profile tests or for diagnostic surveillance programs. Only residual serum and EDTA blood was used in this study.

### 2.2. Immunofluorescence Antibody Test (IFAT)

Specific IgG antibodies to *L. infantum* were detected using IFAT following the OIE Terrestrial Manual—Leishmaniosis protocol [36]. *Leishmania* promastigotes (WHO strain: MHOM/IT/80/IPT1) were used as an antigen, fixed on multispot microscope slides (Bio-Merieux, Marcy L’Etoile, France) in an acetone bath. The feline sera were prepared by serial 2-fold dilutions (1:40 to 1:5120) in phosphate buffered saline (PBS; pH 7.2) and added to the antigen-coated wells. The slides were incubated for 30 min at 37 °C. Positive and negative controls were included in each series of analyzed samples. Fluoresceinated anti-cat IgG antibody (working anti-feline Anti-Cat IgG (whole molecule)—FITC antibody produced in goat, Sigma Aldrich, St. Louis, MO, USA) was used (dilution 1:200 in PBS). The slides were examined using a Leica DM 4000B fluorescence microscope (Leica, Heerbrugg, Switzerland). The cut-off value for positivity was set at 1:80, according to the most recent recommendations on feline leishmaniosis [2] and on a study relative to diagnostic performance of ELISA, IFAT and Western blot for the detection of anti-*L. infantum* antibodies in cats [37].

### 2.3. Real-Time PCR (qPCR)

DNA was extracted from EDTA whole using a PureLink™ Genomic DNA Mini Kit (Thermo Fisher Scientific K182002, Waltham, MA, USA), following the manufacturer’s instructions. The real-time PCR was performed in a QuantStudio 3 (Life Technology, Waltham, MA, USA) and carried out as described by Castelli et al. [38]. RT PCR was conducted in 20 µL reactions containing 10 µL of SsoAdvanced Universal Probes Supermix (Biorad, Hercules, CA, USA), 0.25 µM QLeish Probe, 0.3 µM of each primer and 2 µL of extracted DNA at 10 ng/µL. A preparation of 10-fold serially diluted *L. infantum* parasite DNA corresponding to 1 × 10^6^ to 1 parasite per mL was used as a standard curve. The thermal cycle was set as follows: initial denaturation for 10 min at 95 °C, 40 cycles of denaturation for 15 s at 95 °C and annealing polymerization for 35 s at 60 °C.

### 2.4. Statistical Analysis

Statistical analysis was performed using a commercially available software program (MedCalc Statistical Software version 20.106, MedCalc Software Ltd., Ostend, Belgium). The data were analyzed using standard descriptive statistics. Categorical data were reported as numbers and percentages, while numerical data (age, IFAT titer, DNA parasite load) were reported as mean ± standard deviation (SD) or median and range, depending on whether or not the data were normally distributed. Pearson’s correlation between quantitative load of *Leishmania* DNA (both for qPCR on blood and on lymph node) and IFAT titers were calculated. To identify possible risk and protective factors associated with *L. infantum* infection, a univariate analysis was conducted and odds ratios (OR) with 95% confidence intervals (95% CI) were calculated for each variable statistically significant linked to the infective status. Associations were described using a probability *p*-Value < 0.05 as statistically significant.

## 3. Results

### 3.1. Overall Infection Prevalence

Out of 1490 cats tested, 124 (8.3%, 95% CI 6.9–9.9) scored positive to *L. infantum*. Of these, 96 out of 1460 cats tested with IFAT (6.6%) had specific *L. infantum* IgG, 18 out of 814 tested for parasite DNA (2.2%) were qPCR positive on blood and/or on lymph node, and 10 out of 784 cats tested with both methods (1.3%) were both IFAT and qPCR positive on blood and/or on lymph node. 

**Figure 1:**
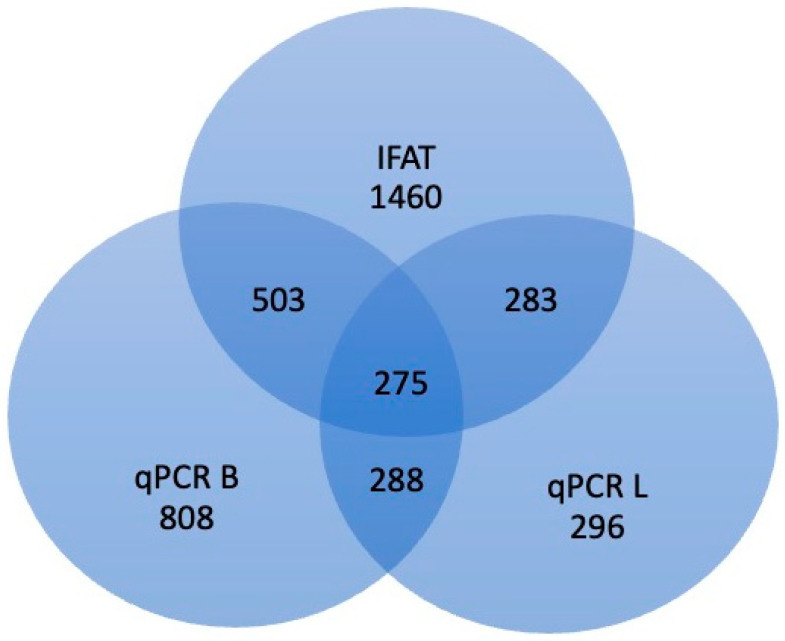
Number of samples tested for *Leishmania infantum* infection using different methods in feline populations from three Italian regions. qPCR B: qPCR on blood; qPCR L: qPCR on lymph node.

Table 1 reports *L. infantum* specific test results in feline populations divided by origin from the three Italian regions evaluated, and Figure 1 reports the number of samples tested by different methods.

**Figure 2:**
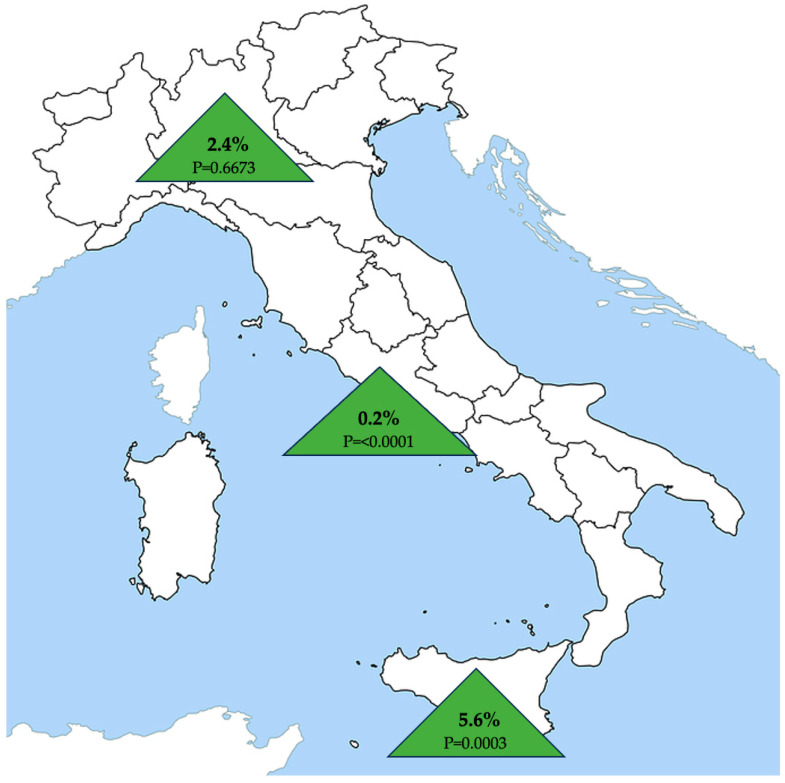
Prevalence of *Leishmania infantum* infection in cats from the Lombardy, Lazio and Sicily regions in northern, central and southern Italy, respectively.

The characteristics of the feline population evaluated and results of univariate analysis of factors potentially associated with the infection are reported in Table 2. Exact age was available for 747 cats that had a median of 2 years (range 2 months-20 years, 25°–75° percentile 1–3.7 years). The 39 pure breed cats were 9 Siamese, 6 Persians, 5 British shorthair, 5 Exotic shorthair, 3 Ragdolls, 3 Siberians, 3 Scottish Folds, 2 Holy Birman, 2 Maine Coon and 1 Bengal. Figure 2 summarizes prevalence of infection in the three Italian regions.

Risk factors for overall infection were: origin from the Sicily region (OR = 2.0, 95% CI 1.3–3.0), male gender (OR = 1.8, 95% CI 1.1–3.2), outdoor lifestyle (OR = 2.3, 95%CI 0.9–5.6) and seropositivity for FIV antibodies (OR = 2.2, 95% CI 1.2–4.2), while origin from the Lazio region (OR = 0.1, 95% CI 0.05–0.4) was a protective factor for infective status. A significant association was found between *L. infantum* overall infection and sampling in winter (OR = 3.2, 95% CI 2.2–4.8), spring (OR = 0.5, 95% CI 0.3–0.7) and summer (OR = 0.3, 95% CI 0.1–0.7).

**Figure 3:**
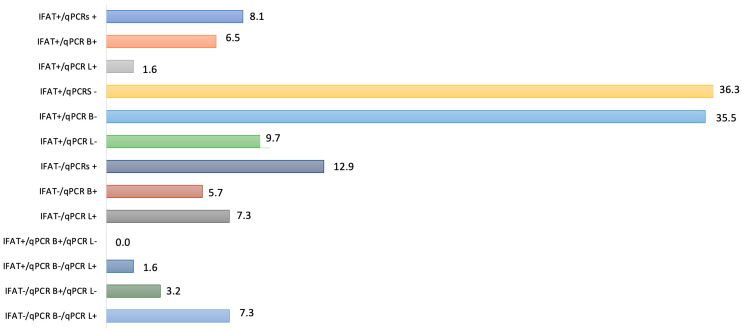
Percentages of positive results according to more than one assay in 124 *Leishmania infantum* infected cats from Italy. B: blood; L: lymph node; qPCRs: positive results by combining qPCR on blood and on lymph node.

In Table 3, the 124 *L. infantum* positive samples are divided by results for the three specific exams performed, i.e., IFAT, qPCR on blood and qPCR on popliteal lymph node aspirate. Figure 3 reports percentages of positive results according to more than one assay. 

Overall qPCR positive results and IFAT seropositivity were statistically significantly associated (*p* < 0.0001), both IFAT with qPCR on blood samples (*p* < 0.0001) and IFAT with qPCR positivity on popliteal lymph node aspirates (*p* = 0.0393). Specifically, a qPCR positive cat had an OR = 9.9 (95% CI = 4.2–23) to be also IFAT seropositive, with OR = 17.8 (95% CI = 6.1–51.3) to be IFAT positive for a cat with positive qPCR on blood and OR = 4.8 (95% CI = 0.9–24.7) to be IFAT positive for a cat with positive qPCR on popliteal lymph node aspirate. No significant correlation was found between the antibody titer and the *Leishmania* parasite load (Pearson r: 0.05; *p* = 0.6196).

Results were also analyzed considering only samples tested by IFAT and only by qPCR, as described below.

### 3.2. Seroprevalence

**Figure 4:**
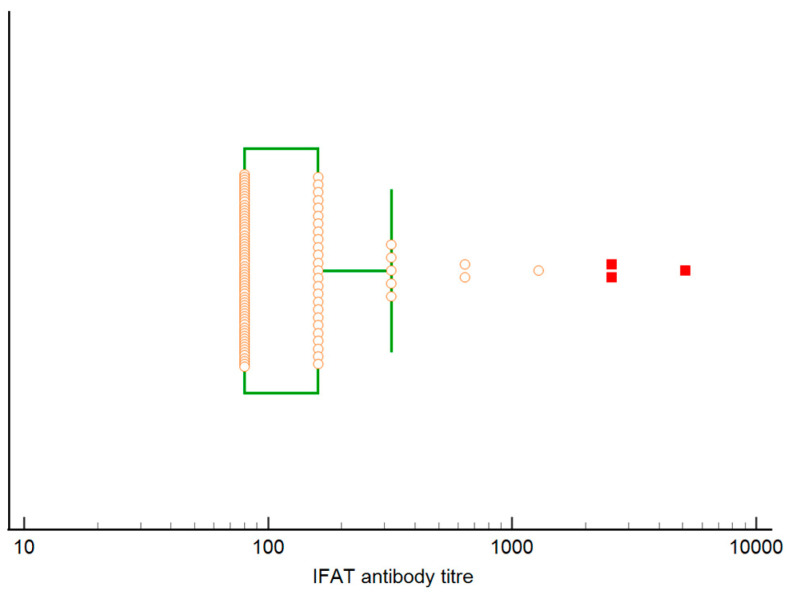
Box and whisker plot reporting *L. infantum* antibody titers in 106 IFAT seropositive cats (circles). The central box represents the values from the 1st to 3rd quartile (25° to 75° percentile), and the median corresponds to 1st quartile. Circles outside the whisker are the outside values, while the far out values are shown as red squares.

Relative to serological analysis, 1354/1460 (92.7%) cats tested by IFAT were seronegative (of which 115 were seroreactive with an antibody titer of 1:40), 106/1460 (7.3%) were seropositive (antibody titer ≥ 1:80), and specifically 70 cats had antibody titers of 1:80, 25 of 1:160, 5 of 1:320, 2 of 1:640, 1 of 1:1280, 2 of 1:2560 and 1 of 1:5120. Median antibody titer was 1:80, with a range 1:80–1:5120, and a 25°–75° percentile of 1:80–1:160 (Figure 4). 

Table 4 reports the characteristics of the feline population analyzed by IFAT and results of univariate analysis of factors statistically significantly associated with seropositivity. Origin from the Sicily region (OR = 2.9, 95% CI 1.8–4.6), FIV seropositivity (OR = 2.1, 95% CI 1.04–4.1), male gender (OR = 1.9, 95% CI 1.02–3.5) and adult age (OR = 2.6, 95% CI 1.1–5.8) were risk factors for seropositivity for specific *L. infantum* antibodies, while origin from the Lombardy (OR = 0.6, 95% CI 0.3–0.9) and Lazio (OR = 0.17, 95% CI 0.06–0.4) regions were protective factors for *L. infantum* seropositivity. A significant association was found between *L. infantum* infection detected by IFAT and sampling in the winter (OR = 4.3, 95% CI 2.8–6.5), spring (OR = 0.6, 95% CI 0.4–0.9) and summer (OR = 0.3, 95% CI 0.1–0.7) seasons.

### 3.3. qPCR Prevalence

**Figure 5:**
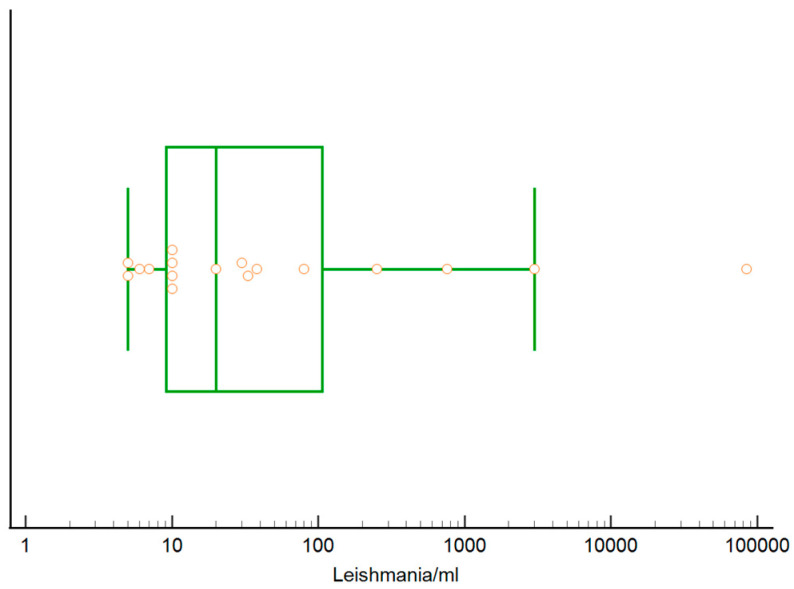
Box and whisker plot reporting *L. infantum* DNA load in blood from 17 qPCR positive cats (circles). The central box represents the values from the lower to upper quartile (25° to 75° percentile), the middle line represents the median. The horizontal line extends from the minimum to the maximum value, excluding the outlier value which is displayed as separate point.

**Figure 6:**
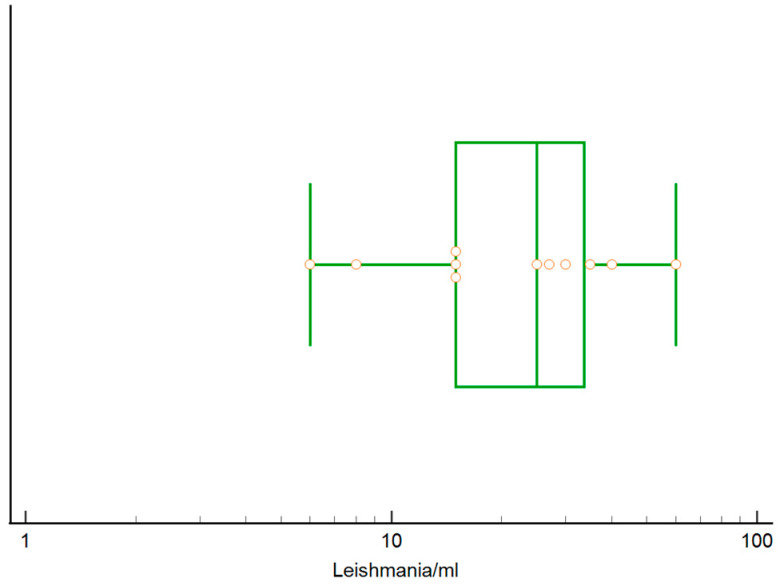
Box and whisker plot reporting *L. infantum* DNA load in popliteal lymph node aspirates of 11 qPCR positive cats (circles). The central box represents the values from the lower to upper quartile (25° to 75° percentile), the middle line represents the median. The horizontal line extends from the minimum to the maximum value.

Results were analyzed only considering qPCR analysis, as reported in Table 5. A total of 814 samples were analyzed by qPCR and 28 (3.4%) were positive. Out of 808 blood samples analyzed, 17 (2.1%) were qPCR positive, while of 296 lymph node aspirates analyzed, 11 (3.7%) were positive. Median parasite DNA content in blood was 20 *Leishmania*/mL (range: 5–84,400, 25°–75° percentile: 9–123 *Leishmania*/mL, Figure 5), while median DNA parasite load in popliteal lymph node aspirate was 25 *Leishmania*/mL (range 6–60 *Leishmania*/mL, 25°–75° percentile 15–34 *Leishmania*/mL, Figure 6). 

Outdoor lifestyle (OR = 15.8, 95% CI 0.9–264.3) was a risk factor for a positive qPCR, while being an owned (OR = 0.2, 95% CI 0.04–0.9), or indoor (OR = 0.1, 95% CI 0.006–1.7) cat was a protective factor. A significant association was found between *L. infantum* infection detected by qPCR and sampling in autumn (OR = 7.03, 95% CI 3.05–16.2) and in spring (OR = 0.2, 95% CI 0.08–0.6).

## 4. Discussion

Leishmaniosis in domestic cats (*Felis catus*) was first described in 1912 in Algeria in a cat living with a dog and a child, both infected with *L. infantum* [39]. Since then, FeL has been reported worldwide but is most frequently found in Mediterranean areas, where it is still considered an emerging feline disease. In cats, the antibody prevalence against *L. infantum* in the Mediterranean area varies from 1.29% to 60% in client-owned cats from central and southern Spain, respectively [12,40], while prevalence of positive PCR on blood varies between 0% in colony stray cats in northern Italy [15] and in central Spain [41] to 28.36% in client-owned cats from northwestern Italy [11]. Therefore *L. infantum* infection prevalence in cats is subject to great variability across geographical areas and this variability in the data depends on the different methods used for FeL diagnosis and on the type of the feline population studied. Furthermore, the diagnostic methods employed often differ in protocols, for example, using different cut-off values for the serological tests for specific *L. infantum* antibodies or for different biological samples or PCR targets used. Finally, serological and molecular tests are rarely used simultaneously in the same animal population for the diagnosis of FeL, limiting the overall information on the presence of infection in the surveyed cats.

In Italy, leishmaniosis is considered endemic in south and central Italy and in the islands. In the last 20 years, sand fly vectors as well as human and canine *Leishmania* infections have been detected in northern Italy, traditionally classified as a cold area unsuitable for sand fly survival [42,43]. Today, human and animal leishmaniosis is endemic throughout the Italian peninsula, showing an increasing gradient from north to south and showing the highest incidence in Sicily [44,45]. Bruno et al. [45] used a One Health approach, integrating human and animal data, to assess and map the risk of endemic transmission of *Leishmania* in Sicily, and found positive associations between human cases, infected reservoir hosts and vector spatial distribution.

Data showed that, in the north of the country the number of dogs and cats that are seropositive for *L. infantum* has increased in the last decade, showing a progressive spread from the endemic southern regions towards the northern regions, making the entire Italian peninsula endemic for *Leishmania* infection today [42,43,44,46]. Two previously published studies have already evaluated cats from different regions in Italy for *L. infantum* infection [17,19]. The first one, by Iatta et al., was published in 2019 [17] and showed both serological and molecular prevalence lower than our results, of 3.3% (88/2659) and 0.8% (22/2659) respectively. Although that study was conducted, like ours, on cats from northern, central and southern Italy, the lack of the PCR examination on lymph nodes may have resulted in an underestimation of the infection prevalence. Furthermore, the cats recruited in that study were all owned cats which, unlike stray and shelter feline populations, are less exposed to vector ectoparasites, especially in highly endemic areas [9]. The second nationwide published study was a molecular survey of vector-borne pathogens and hemoplasmas in owned cats across Italy that was published one year later, in 2020 [19]. In that study, the DNA molecular prevalence for *L. infantum* in blood samples was 3.2% (31/958), therefore higher than our molecular prevalence of 2.1% in blood samples. This difference can be explained by the fact that, in that study, feline blood samples came from veterinary analytical laboratories and were collected during an animal’s health check-up, making it more likely that samples were from unhealthy cats and increasing the possibility that these cats would be *Leishmania* infected. 

Stray colony cats from urban areas of the city of Milan and the suburbs of the Lombardy region in northern Italy have been monitored for *L. infantum* infection by our research group since in 2008, with the publication of three epidemiological studies [15,16,18]. Since the present study evaluates 229 cats from a stray population in the suburbs of Milan, we can compare the seroprevalence rate between these studies. This shows a decrease from 9.0% (21/233) in the years 2008–2010 [15] to 5.5% (12/218) in the current study. Conversely, the PCR detection rate on blood samples increased from 0% (0/233) in 2008–2010 [15] to 2.2% (5/225) in this study, or to 5.2% if we add the results of qPCR analysis on lymph nodes (7 cats positive from 229 analyzed). Lymph nodes were also evaluated in our previous study with a prevalence of infection increase from 1.1% [16] in 2014, to 4.4% in 2016–2018 [18]. The spread of leishmaniosis in Italy, as in the rest of Europe, depends on a number of factors, including global warming, that affect the ecology and distribution of phlebotomine vectors, and anthropogenic risk factors such as migration, travel, and animal trade [47]. Therefore, another possible explanation for the increasing molecular prevalence of infection in the northern Italian feline population investigated in our study could be the spread of the sandfly vectors in the northern region of Italy in the last few years as a result of climate change. This has been shown by different entomological studies that have documented sandfly vectors of *Leishmania* in many areas of northern Italy [42,48]. It should also be remembered that some studies [49,50] have identified the *L. infantum* genome in ectoparasites such as ticks. Ticks are widely spread in stray feline populations, which in Italy do not receive any prophylaxis against ecto- and endoparasites. However, the role of ticks in the transmission of *Leishmania* has yet to be fully determined.

The Italian regional incidence of human leishmaniosis cases in 2011–2016 was above the national average (0.70 cases per 100,000 population) in the Sicily (1.93), Liguria (1.59), the island of Sardinia (1.09), Emilia–Romagna (1.04), Lazio (0.84), Campania (0.80), Calabria (0.78) and Tuscany (0.74) regions [5]. Given these data, it is surprising that cats from the Lazio region evaluated in our study showed the lowest seroprevalence for antibodies against *L. infantum* and origin from this Italian region was a protective factor for infective status. In central Italy, feline leishmaniosis has been poorly investigated and only recently, in a congress abstract published in 2022 [51], the FeL epidemiology of the Lazio region was evaluated, testing a total of 200 feline serum and blood samples collected from cats admitted at a veterinary research laboratory as routine controls. Specific IgG antibodies against *L. infantum* were found in 4 of 200 (2%) tested cats, with antibody titers ranging from 1:40 (3/200) to 1:80 (1/200). Therefore, only one cat showed an antibody titer equal to 1:80, the cut-off for seropositivity used in our study, with an overall seroprevalence of 0.5%. This seroprevalence is lower than in our data, but together with our results confirmed a low seroprevalence of the infection in cats from this region. Molecular investigations in this population are still ongoing and will serve to better clarify the epidemiological picture of FeL in Lazio and to confirm whether the infection is really present at such a low prevalence in this region. 

Our study confirmed the highest prevalence of FeL in the Sicily region in southern Italy, as reported by recent published studies [9,45,52]. This region has the highest endemicity for animal leishmaniosis and highest incidence of human infection among the Italian regions [5,45]. Originating from the Sicily region was a risk factor for seropositivity for antibodies against *L. infantum*, but not for qPCR positivity. This can be explained by the fact that cats from Sicily were not surveyed for parasite DNA in lymph node samples, which is a more sensitive test for detection of *Leishmania* DNA in infected cats and in other species such as dogs [53,54]. 

Several studies in recent years on FeL in cats have analyzed various risk factors that could increase the susceptibility to infection in this species. Among the factors investigated, most research has been done on the co-infections of *Leishmania* spp. and other pathogens, and among those found to be significant are infections with *Toxoplasma gondii* and *Neospora caninum* [55], feline coronavirus (FCoV) [16], hemotropic mycoplasmas [11], FIV [15,22,23,24,56] and FeLV [12,24,25]. FIV seropositivity was found to be a risk factor for FeL infection in our study, but only for overall infective status and seropositivity for *L. infantum* antibodies, and not for the presence of parasite DNA. FIV seropositive cats had a 2.1-fold higher risk of being *L. infantum* antibody positive. The significant association between *L. infantum* and FIV infection found in our study has previously been reported [11,15,17,22,23,24,56,57,58]. In particular, as previously demonstrated [57], in our study FIV seropositive cats were more likely to be *L. infantum* seropositive at IFAT, while the same is not true if we consider only *Leishmania* qPCR positivity. This could be due to the lower number of qPCR-positive cats (n = 28) when compared to IFAT positive samples (n = 106). Consequently, results concerning association between FIV and qPCR in our study should be interpreted with caution, and a larger sample size should be assessed to confirm the association that was evident in previous studies [17,56]. The association in this study between FIV and *L. infantum* seropositivity confirms that immunosuppressive agents, such as the FIV virus, could impair the cellular immune response, thereby increasing the risk of contracting the infection and developing clinical signs of FeL. FIV and *L. infantum* co-infections could predispose animals to visceral forms or to more generalized forms of the infection, as a consequence of viral immunosuppression, exactly as recognized in HIV seropositive patients [59]. The protective immune response against *Leishmania* parasites is mediated by CD4+ T helper1 (Th1) lymphocytes. Consequently, all agents that induce suppression of the CD4+ T lymphocyte-mediated immune response, such as FIV infection, can be considered risk factors for *Leishmania* infection. However, it should be borne in mind that the presence of FIV alone is not a sufficient marker to demonstrate immunodeficient status; this requires additional immunological tests. In this study, the stage of FIV infection was not assessed with immunological markers, and this is a limitation shared with all field studies that have so far investigated the association between FIV and *L. infantum* infection [57]. Adaptive humoral and cell-mediated immune response is elicited by *L. infantum* feline infection, but no difference was found in FIV seropositive cats in a study evaluating the ex vivo blood production of *L. infantum*- specific IFN-γ [60].

Sampling in winter was significantly associated with a higher overall *L. infantum* infection prevalence, in particular for seropositive cats, and this can be explained by the time necessary to mount detectable antibodies levels in cats infected during the season of activity of the sandfly vectors. Sampling in autumn was instead associated with qPCR positivity, and this could be due to the presence of parasite DNA soon after the sandfly bite. On the contrary, significantly fewer cats were *L. infantum* seropositive in spring and summer, and fewer were qPCR positive when sampled in spring. Again, these results could arise from the fact that the activity of sand fly vectors begins in spring and summer, therefore cats could not already have mounted a detectable antibody level nor have parasite DNA in blood or in the reticuloendothelial system in these seasons. However, this explanation is only speculative, as we do not know when the cats in our study contracted the infection during the four year time frame of investigation and we have no information on the distribution of sand fly vectors during the years and months in the studied areas.

Male gender was a risk factor for overall and, in particular, for IFAT positivity; adult age was a risk factor only for IFAT seropositivity, while outdoor lifestyle was a risk factor for qPCR positivity. Adult age has previously been reported as a risk factor for feline *L. infantum* positivity [24,56,61,62], with adult cats being more frequently infected. Males were positive more often than females in other studies detecting a significant sex difference in *L. infantum* positivity of cats [23,62,63]. These factors demonstrate the importance of increased frequency and time of the exposure to environments where the sandfly vector is found in endemic areas where no preventative measures are implemented. This could represent a risk factor that increases the risk for *Leishmania* infection. In fact, to be an owned indoor cat was a protective factor for parasitemia and qPCR positivity.

Neither the clinical status nor any of the clinical signs considered in our study were associated with infective status and this could be due to the fact that most infected cats were asymptomatic [1,11].

A significant association was found between IFAT and qPCR positivity in infected cats, which shows that cats that test positive for qPCR have an 11-fold greater chance of being seropositive for *L. infantum* IgG on the IFAT test. This association was significant only for blood PCR (OR = 20.0, 95% CI = 6.6–60.5, *p* < 0.0001). Therefore, cats with generalized infection, i.e., those in which *Leishmania* parasites were found in the reticuloendothelial system, such as in lymph node tissue, were not always seropositive for antibodies against *Leishmania*. This underlines the point that as many techniques as possible should be applied to identify infected subjects. When molecular techniques such as PCR are used, these should be applied to as many substrates as possible, and in particular those with high sensitivity for detecting infection, such as lymph node and/or bone marrow [54]. However, in contrast to what was previously found in canine leishmaniosis [53], no significant correlation was found between IFAT antibody titer and parasite load at qPCR, and this could due to the small numbers of cats that were tested and found to be positive with qPCR in blood and on lymph node.

This study has some limitations. The first one is that large cross-sectional studies such as our study are often based on routinely collected samples. This means that history and clinical findings data can be incomplete and do not provide information on some confounding factors. The lack of some data precluded the study of some factors associated with the infection, most notably in feline stray colony populations in which history is often lacking. The absence of PCR analysis on the samples from the Lazio region, as well as the failure to analyze the lymph node tissue in Sicilian cats with PCR techniques, may have led to an underestimation of the infection prevalence in these regions. The lack of molecular methods in some cats may not have allowed the diagnosis of infection, since resistant animals, or those in the first phase of the infection, will be negative to the IFAT test, but could have a positive PCR test [10]. In addition, lack of many data on the same feline population precluded the possibility of running a multivariate logistic regression analysis of factors that were associated with *L. infantum* infection at univariate analysis. Finally, we did not consider other covariables that could have affected our results, such as the presence of *Leishmania* reservoirs and vectors and human and canine cases of leishmaniosis.

## 5. Conclusions

As previously reported [64], we found that when multiple tests are used in endemic areas to assess the prevalence of infection with *L. infantum* in cats, the combination of results from molecular and serological methods increases diagnostic sensitivity. Therefore, both molecular and serological tests should be performed for the diagnosis of FeL, especially in endemic areas, regardless of the presence or absence of clinical signs. To reduce the risk of *Leishmania* infection in feline populations in endemic areas, cats should be protected against the bite of sandfly vectors, which is of particular importance in FIV infected cats.

## Figures and Tables

**Table 1:** Specific *Leishmania infantum* diagnostic tests performed in different feline populations from three Italian regions.

	Lombardy n Positive/Total Tested (%)	Lazio n (%)	Sicily n (%)	Total n (%)
**IFAT**	22/431 (5.1%)	4/257 (1.6%)	80/772 (10.4%)	106/1460 (7.3%)
**qPCR on blood**	6/443 (1.4%)	Not performed	11/365 (3.0%)	17/808 (2.1%)
**qPCR on lymph node**	11/296 (3.7%)	Not performed	Not performed	11/296 (3.7%)

IFAT: indirect fluorescent antibody test; qPCR: quantitative polymerase chain reaction (real-time PCR).

**Table 2:** Descriptive statistics of categorical variables relative to different feline populations from Italy for cats infected and uninfected with *Leishmania infantum*. In bold are significant *p*-Values (<0.05).

Parameter n = Number of Subjects for Which Data Were Available	Variables	*L. infantum* Infected n. (%)	*L. infantum* Uninfected n. (%)	*x*^2^ and *p*-Value
Sampling season n = 1485	Spring	42 (2.8)	668 (44.9)	*x*^2^ = 10.58 ***p* = 0.0012**
	Summer	8 (0.5)	222 (14.9)	*x*^2^ = 8.4 ***p* = 0.0037**
	Autumn	28 (1.8)	264 (17.7)	*x*^2^ = 0.7 *p* = 0.3934
	Winter	46 (3.1)	207 (13.9)	*x*^2^ = 38.4 ***p* < 0.0001**
Region of origin n = 1490	Lombardy	36 (2.4)	422 (28.3)	*x*^2^ = 0.18 *p* = 0.6673
	Lazio	4 (0.2)	253 (16.9)	*x*^2^ = 18.6 ***p* < 0.0001**
	Sicily	84 (5.6)	691 (46.3)	*x*^2^ = 13.3 ***p* = 0.0003**
Habitat n = 1004	Stray	37 (3.6)	633 (63.0)	*x*^2^ = 1.48 *p* = 0.2237
	Shelter	10 (1.0)	87 (8.6)	*x*^2^ = 3.16 *p* = 0.0753
	Owned	15 (1.4)	222 (22.1)	*x*^2^ = 0.013 *p* = 0.9104
Breed n = 789	European	43 (5.4)	707 (89.6)	*x*^2^ = 0.25 *p* = 0.6109
	Other	3 (0.3)	36 (4.5)	
Hair length n = 789	Short hair	44 (5.5)	718 (91.0)	*x*^2^ = 0.1 *p* = 0.7221
	Medium/long hair	2 (0.2)	25 (3.1)	
Gender n = 995	Male	35 (3.5)	407 (40.9)	*x*^2^ = 5.6 ***p* = 0.0176**
	Female	24 (2.4)	529 (53.1)	
Reproductive status n = 995	Neutered	13 (1.3)	206 (20.7)	*x*^2^ = 0.00 *p* = 0.9964
	Intact	46 (4.6)	730 (73.3)	
Age n = 919	Kitten	14 (1.5)	324 (35.2)	*x*^2^ = 1.49 *p* = 0.2210
	Adult	33 (3.5)	466 (50.7)	*x*^2^ = 3.5 *p* = 0.0596
	Senior	2 (0.2)	80 (8.7)	*x*^2^ = 1.49 *p* = 0.2220
Lifestyle n = 600	Indoor	4 (0.6)	99 (16.5)	*x*^2^ = 2.5 *p* = 0.1131
	Outdoor	40 (6.6)	409 (68.1)	*x*^2^ = 3.8 ***p* = 0.0488**
	Indoor/outdoor	2 (0.3)	46 (7.6)	*x*^2^ = 0.9 *p* = 0.3424
Prophylaxis against ectoparasites n = 537	Regular	5 (0.9)	82 (15.2)	*x*^2^ = 0.7 *p* = 0.3967
	Irregular	10 (1.8)	256 (47.6)	*x*^2^ = 0.15 *p* = 0.6962
	None	7 (1.3)	177 (32.9)	*x*^2^ = 0.06 *p* = 0.8052
Cohabitation with dogs n = 827	Yes	2 (0.2)	15 (1.8)	*x*^2^ = 0.8 *p* = 0.3625
	No	51 (6.1)	759 (91.7)	
Moving from the place of residence n = 408	Yes	3 (0.7)	75 (18.3)	*x*^2^ = 0.7 *p* = 0.3762
	No	7(1.7)	323 (79.1)	
Unhealthy n = 533	Yes	5 (0.9)	106 (19.8)	*x*^2^ = 0.2 *p* = 0.6396
	No	15 (2.8)	407 (76.3)	
Clinical status n = 533	Weight loss n = 374	3 (0.8)	46 (12.3)	*x*^2^ = 0.6 *p* = 0.4196
	Lymphadenomegaly n = 523	2 (0.3)	19 (3.6)	*x*^2^ = 2.16 *p* = 0.1412
	Cutaneous signs n = 524	3 (0.5)	53 (10.1)	*x*^2^ = 0.5 *p* = 0.4638
	Ocular signs n = 527	0 (0.0)	39 (7.4)	*x*^2^ = 1.5 *p* = 0.2099
FIV n = 1024	Seropositive	14 (1.3)	82 (8.0)	*x*^2^ = 6.7 ***p* = 0.0094**
	Seronegative	66 (6.4)	862 (84.1)	
FeLV n = 1024	Seropositive	7 (0.6)	57 (5.5)	*x*^2^ = 0.9 *p* = 0.3362
	Seronegative	73 (7.1)	887 (86.6)	
FIV + FeLV n = 1024	Seropositive	2 (0.2)	7 (0.6)	*x*^2^ = 2.6 *p* = 0.1058
	Seronegative	78 (7.6)	937 (91.5)	

FIV: feline immunodeficiency virus; FeLV: feline leukemia virus.

**Table 3:** Results of different tests performed in 124 *Leishmania infantum* infected cats from Italy.

Positivity to Tests for *L. infantum*	n	%
Only IFAT	96	77.4
IFAT + qPCR on lymph node	2	1.5
IFAT + qPCR on blood	8	6.5
Only qPCR on lymph node	9	7.3
Only qPCR on blood	9	7.3
qPCR on blood + on lymph node	0	0.0

IFAT: Indirect fluorescent antibody test; qPCR: quantitative real-time polymerase chain reaction.

**Table 4:** Descriptive statistics of categorical variables of 1460 cats from different feline populations in Italy that were seropositive and seronegative at indirect fluorescent antibody test (IFAT) for specific *Leishmania infantum* antibodies. In bold are significant *p*-Values (<0.05).

Parameter n = Number of Subjects for Which Data Were Available	Variables	IFAT Seropositive n. (%)	IFAT Seronegative n. (%)	*x*^2^ and *p*-Value
Sampling season n = 1455	Spring	39 (2.6)	662 (45.5)	*x*^2^ = 5.9 ***p* = 0.0149**
	Summer	6 (0.4)	221 (15.1)	*x*^2^ = 8.5 ***p* = 0.0034**
	Autumn	16 (1.1)	270 (18.5)	*x*^2^ = 1.5 *p* = 0.2198
	Winter	45 (3.0)	196 (13.4)	*x*^2^ = 55.4 ***p* < 0.0001**
Region of origin n = 1460	Lombardy	22 (1.5)	409 (28.0)	*x*^2^ = 4.2 ***p* = 0.0400**
	Lazio	4 (0.2)	253 (17.3)	*x*^2^ = 15.06 ***p* = 0.0001**
	Sicily	80 (5.4)	692 (47.4)	*x*^2^ = 23.4 ***p* < 0.0001**
Habitat n = 975	Stray	24 (2.4)	635 (65.1)	*x*^2^ = 3.5 *p* = 0.0586
	Shelter	6 (0.6)	89 (9.1)	*x*^2^ = 0.7 *p* = 0.3731
	Owned	14 (1.4)	207 (21.2)	*x*^2^ = 2.1 *p* = 0.1381
Breed n = 760	European	28 (3.6)	698 (91.9)	*x*^2^ = 0.3 *p* = 0.5535
	Other	2 (0.2)	32 (4.2)	
Hair length n = 760	Short hair	28 (3.6)	706 (92.8)	*x*^2^ = 0.994 *p* = 0.3187
	Medium/long hair	2 (0.2)	24 (3.1)	
Gender n = 968	Male	25 (2.5)	402 (41.5)	*x*^2^ = 4.2 ***p* = 0.0398**
	Female	17 (1.7)	524 (54.1)	
Reproductive status n = 968	Neutered	9 (0.9)	199 (20.5)	*x*^2^ = 0.0 *p* = 0.9924
	Intact	33 (3.4)	727 (75.1)	
Age n = 891	Kitten	7 (0.7)	321 (36.0)	*x*^2^ = 3.1 *p* = 0.0745
	Adult	24 (2.6)	460 (51.3)	*x*^2^ = 5.7 ***p* = 0.0168**
	Senior	1 (0.11)	78 (8.7)	*x*^2^ = 1.3 *p* = 0.2448
Lifestyle n = 575	Indoor	4 (0.7)	88 (15.3)	*x*^2^ = 0.3 *p* = 0.5317
	Outdoor	27 (4.7)	411 (71.4)	*x*^2^ = 0.6 *p* = 0.4335
	Indoor/outdoor	2 (0.3)	43 (7.4)	*x*^2^ = 0.15 *p* = 0.6976
Prophylaxis against ectoparasites n = 512	Regular	5 (0.9)	77 (15.0)	*x*^2^ = 1.9 *p* = 0.1664
	Irregular	7 (1.3)	250 (48.8)	*x*^2^ = 0.9 *p* = 0.3292
	None	6 (1.1)	167 (32.6)	*x*^2^ = 0.00 *p* = 0.9668
Cohabitation with dogs n = 805	Yes	2 (0.2)	13 (1.6)	*x*^2^ = 2.9 *p* = 0.0852
	No	33 (4.1)	757 (94.0)	
Moving from the place of residence n = 394	Yes	3 (0.7)	69 (17.5)	*x*^2^ = 0.9 *p* = 0.3317
	No	7 (1.7)	315 (79.9)	
Unhealthy n = 512	Yes	4 (0.7)	100 (19.5)	*x*^2^ = 0.2 *p* = 0.6362
	No	12 (2.3)	396 (77.3)	
Clinical status n = 512	Weight loss n = 358	2 (0.5)	45 (12.5)	*x*^2^ = 0.06 *p* = 0.8064
	Lymphadenomegaly n = 502	2 (0.4)	19 (3.7)	*x*^2^ = 2.8 *p* = 0.0916
	Cutaneous signs n = 503	3 (0.6)	50 (9.9)	*x*^2^ = 1.1 *p* = 0.2773
	Ocular signs n = 506	0 (0.0)	36 (7.1)	*x*^2^ = 1.2 *p* = 0.2611
FIV n = 1001	Seropositive	11 (1.1)	85 (8.4)	*x*^2^ = 4.5 ***p* = 0.0330**
	Seronegative	53 (5.2)	852 (85.1)	
FeLV n = 1001	Seropositive	6 (0.6)	58 (5.7)	*x*^2^ = 1.0 *p* = 0.3138
	Seronegative	58 (5.7)	879 (87.8)	
FIV + FeLV n = 1001	Seropositive	2 (0.2)	7 (0.7)	*x*^2^ = 3.7 *p* = 0.0513
	Seronegative	62 (6.1)	930 (92.9)	

FIV: feline immunodeficiency virus; FeLV: feline leukemia virus.

**Table 5:** Descriptive statistics of categorical variables relative to 814 cats belonging to different feline populations in Italy that were positive and negative for *Leishmania infantum* DNA tested with quantitative PCR (qPCR, real-time PCR) on blood and/or on popliteal lymph node aspirate. In bold are significant *p*-Values (<0.05).

Parameter n = Number of Subjects for Which Data Were Available	Variables	qPCR Positive n. (%)	qPCR Negative n. (%)	*x*^2^ and *p*-Value
Sampling season n = 814	Spring	4 (0.4)	326 (40.0)	*x*^2^ = 8.2 *p* = 0.0040
	Summer	2 (0.2)	140 (17.2)	*x*^2^ = 2.1 *p* = 0.1440
	Autumn	20 (2.4)	206 (25.3)	*x*^2^ = 27.5 *p* < 0.0001
	Winter	2 (0.2)	114 (14.0)	*x*^2^ = 1.1 *p* = 0.2738
Region of origin n = 814	Lombardy	17 (2.0)	432 (53.0)	*x*^2^ = 0.3 *p* = 0.5478
	Lazio	0 (0.0)	0 (0.0)	*-*
	Sicily	11 (1.3)	354 (43.4)	*x*^2^ = 0.3 *p* = 0.5478
Habitat n = 629	Stray	18 (2.8)	345 (54.8)	*x*^2^ = 3.0 *p* = 0.0807
	Shelter	4 (0.6)	80 (12.7)	*x*^2^ = 0.2 *p* = 0.6270
	Owned	2 (0.3)	180 (28.6)	*x*^2^ = 5.1 *p* = 0.0234
Breed n = 488	European	18 (3.6)	432 (88.5)	*x*^2^ = 0.17 *p* = 0.6757
	Other	1 (0.2)	37 (7.5)	
Hair length n = 488	Short hair	19 (3.8)	443 (90.7)	*x*^2^ = 1.1 *p* = 0.2920
	Medium/long hair	0 (0.0)	26 (5.3)	
Gender n = 640	Male	11 (1.7)	307 (47.9)	*x*^2^ = 0.14 *p* = 0.7005
	Female	13 (2.0)	309 (48.2)	
Reproductive status n = 640	Neutered	5 (0.7)	158 (24.6)	*x*^2^ = 0.2 *p* = 0.5955
	Intact	19 (2.9)	458 (71.5)	
Age n = 592	Kitten	9 (1.5)	173 (29.2)	*x*^2^ = 1.9 *p* = 0.1602
	Adult	10 (1.6)	323 (54.5)	*x*^2^ = 0.3 *p* = 0.5668
	Senior	1 (0.1)	76 (12.8)	*x*^2^ = 1.17 *p* = 0.2792
Lifestyle n = 511	Indoor	0 (0.0)	98 (19.1)	*x*^2^ = 4.6 *p* = 0.0306
	Outdoor	19 (3.7)	350 (68.4)	*x*^2^ = 7.5 *p* = 0.0059
	Indoor/outdoor	0 (0.0)	44 (8.6)	*x*^2^ = 1.8 *p* = 0.1731
Prophylaxis against ectoparasites n = 244	Regular	0 (0.0)	54 (22.1)	*x*^2^ = 1.15 *p* = 0.2833
	Irregular	3 (1.2)	122 (50)	*x*^2^ = 0.9 *p* = 0.3386
	None	1 (0.4)	64 (26.2)	*x*^2^ = 0.00 *p* = 0.9405
Cohabitation with dogs n = 547	Yes	0 (0.0)	16 (2.99	*x*^2^ = 0.7 *p* = 0.3954
	No	23 (4.2)	508 (92.8)	
Moving from the place of residence n = 141	Yes	0 (0.0)	42 (29.7)	-
	No	0 (0.0)	99 (70.2)	
Unhealthy n = 303	Yes	1 (0.3)	107 (35.3)	*x*^2^ = 0.5 *p* = 0.4622
	No	4 (1.3)	191 (63.0)	
Clinical status n = 303	Weight loss n = 148	1 (0.6)	46 (31.0)	*x*^2^ = 0.00 *p* = 0.9529
	Lymphadenomegaly n = 295	0 (0.0)	20 (6.7)	*x*^2^ = 0.2 *p* = 0.5877
	Cutaneous signs n = 295	0 (0.0)	55 (18.6)	*x*^2^ = 0.9 *p* = 0.3359
	Ocular signs n = 299	0 (0.0)	38 (12.7)	*x*^2^ = 0.5 *p* = 0.4431
FIV n = 664	Seropositive	3 (0.4)	74 (11.1)	*x*^2^ = 0.00 *p* = 0.9925
	Seronegative	23 (3.4)	564 (84.9)	
FeLV n = 664	Seropositive	3 (0.4)	29 (4.3)	*x*^2^ = 2.6 *p* = 0.1029
	Seronegative	23 (3.4)	609 (91.7)	
FIV + FeLV n = 664	Seropositive	0 (0.0)	7 (1.0)	*x*^2^ = 0.2 *p* = 0.5916
	Seronegative	26 (3.9)	631 (95.0)	

FIV: feline immunodeficiency virus; FeLV: feline leukemia virus.

## Data Availability

All data included in this study are available on reasonable request by contacting the corresponding author. Results of this research were presented in part at the 2023 International Society of Feline Medicine (ISFM) Feline Congress, 29 June–2 July 2023, Dublin, Ireland.
